# Deep learning‐based multi‐omics study reveals the polymolecular phenotypic of diabetic kidney disease

**DOI:** 10.1002/ctm2.1301

**Published:** 2023-06-08

**Authors:** Huan Zhao, Yu Yuan, Siyu Chen, Yaqi Yao, Chenghao Bi, Chuanxin Liu, Guijiang Sun, Haihua Su, Xinyue Li, Xiaomeng Li, Xingxu Yan, Yubo Li

**Affiliations:** ^1^ State Key Laboratory of Component‐based Chinese Medicine Tianjin University of Traditional Chinese Medicine Tianjin China; ^2^ Henan Key Laboratory of Rare Diseases Endocrinology and Metabolism Center The First Affiliated Hospital and College of Clinical Medicine of Henan University of Science and Technology Luoyang China; ^3^ School of Chinese Materia Medica Beijing University of Chinese Medicine, Liangxiang Town, Fangshan District Beijing China; ^4^ Second Hospital of Tianjin Medical University Tianjin China; ^5^ Department of Endocrinology and Nephrology PKU Care CNOOC Hospital Tianjin China

Dear Editor,

Approximately 30% to 40% of patients with type 2 diabetes mellitus (T2DM) develop diabetic kidney disease (DKD), and most will go on to develop end‐stage renal disease.^1^ The presence of kidney disease complicates the management of patients with T2DM.^2^ Therefore, identifying biomarkers for the early diagnosis of DKD based on circulating molecular factors associated with physiological alterations in patients with T2DM can effectively reduce and delay the incidence of DKD. We used deep learning (DL) to analyze and process multi‐omics data and establish key molecular characteristics (biomarker panels) that affect the incidence and development of DKD.

Based on strict diagnostic inclusion and exclusion criteria, 405 subjects from two centers in China were included in the discovery (*n* = 105) and test (*n* = 300) sets and divided into healthy control (HC), T2DM, and DKD groups (Table 1 and Supplementary Materials).

**TABLE 1:** Characteristics of the participants included in the discovery set.

Trait	HC	T2DM	DKD	*p*‐Value
**Demographic data**				
*n*	35	35	35	
Age (years)	64.2 ± 9.4	63.5 ± 5.9	64.5 ± 8.9	>.05
Sex (male/female)	18/17	18/17	17/18	>.05
**Blood pressure**				
SBP (mm Hg)	133.9 ± 16.9	139.1 ± 13.7	133.2 ± 13.1	>.05
DBP (mm Hg)	75.7 ± 12.4	81.7 ± 7.0	79.7 ± 10.6	>.05
**Blood lipid index**				
TG (mmol/L)	1.2 ± .3	2.5 ± 2.5**	2.3 ± 3.2**	<.01
TC (mmol/L)	4.7 ± .6	5.3 ± 1.8	4.8 ± 1.1	>.05
HDL (mmol/L)	1.3 ± .1	1.0 ± .3**	1.0 ± .3**	<.01
LDL (mmol/L)	2.8 ± .5	3.9 ± 1.5	3.1 ± .89	>.05
AASI	2.1 ± .6	3.2 ± 1.7**	3.0 ± 1.0**	<.01
**Diabetes index**				
HbA1c (%)	5.6 ± .3	6.9 ± 1.4**	8.0 ± 1.6**	<.01
Glu (mmol/L)	5.4 ± .4	7.5 ± 2.7**	8.7 ± 2.3**	<.01
Malb (mg/24 h)	N/A	N/A	50.8 ± 95.9	
**History**				
Diabetes history (year)	N/A	7.1 ± 5.6	14.6 ± 7.6^##^	<.01
**Kidney function**				
Blood urea nitrogen (BUN) (mmol/L)	5.4 ± 1.3	N/A	14.3 ± 44.6*	<.05
Serum creatinine (SCR) (μmol/L)	64.7 ± 12.1	N/A	79.3 ± 37.6*	<.05

*Note*: Data are mean ± SD for continuous measures and *n* for categorical measure. *: Compared with HC (*p < .05; ** *p* < .01); #: Compared with T2DM (#*p* < .05; ##*p* < .01); AASI, Ambulatory Arterial Stiffness Index; N/A, not Applicable; HC, healthy control group; LDL, Low Density Lipoprotein; T2DM, type 2 diabetes mellitus group; TC, Total Cholesterol; TG, Triglyceride; DKD, diabetic kidney disease group.

In the discovery set, the combination of lipidomics and data‐independent acquisition quantitative proteomics enabled the discovery of additional potential biomarkers and pathological mechanisms related to the occurrence and development of DKD. Lipidomics revealed that the metabolic profile of the both disease group changed significantly compared to that of HC; however, the metabolic profiles of T2DM and DKD groups were relatively similar (Figure 1A). Using the criteria of variable importance in projection > 1 and *p* < .05, 70 differential serum metabolites (Table S2) were identified (Figure 1B and Figure S1A). These mainly involved metabolic pathways, such as sphingolipid metabolism, steroid hormone biosynthesis, glycerol phospholipid metabolism and arachidonic acid metabolism (Figure 1C). In addition, the distribution of lipid abundance and lipid classes among the all groups showed that the glycerolipid and glycerophospholipid proportions were the highest.

**FIGURE 1:**
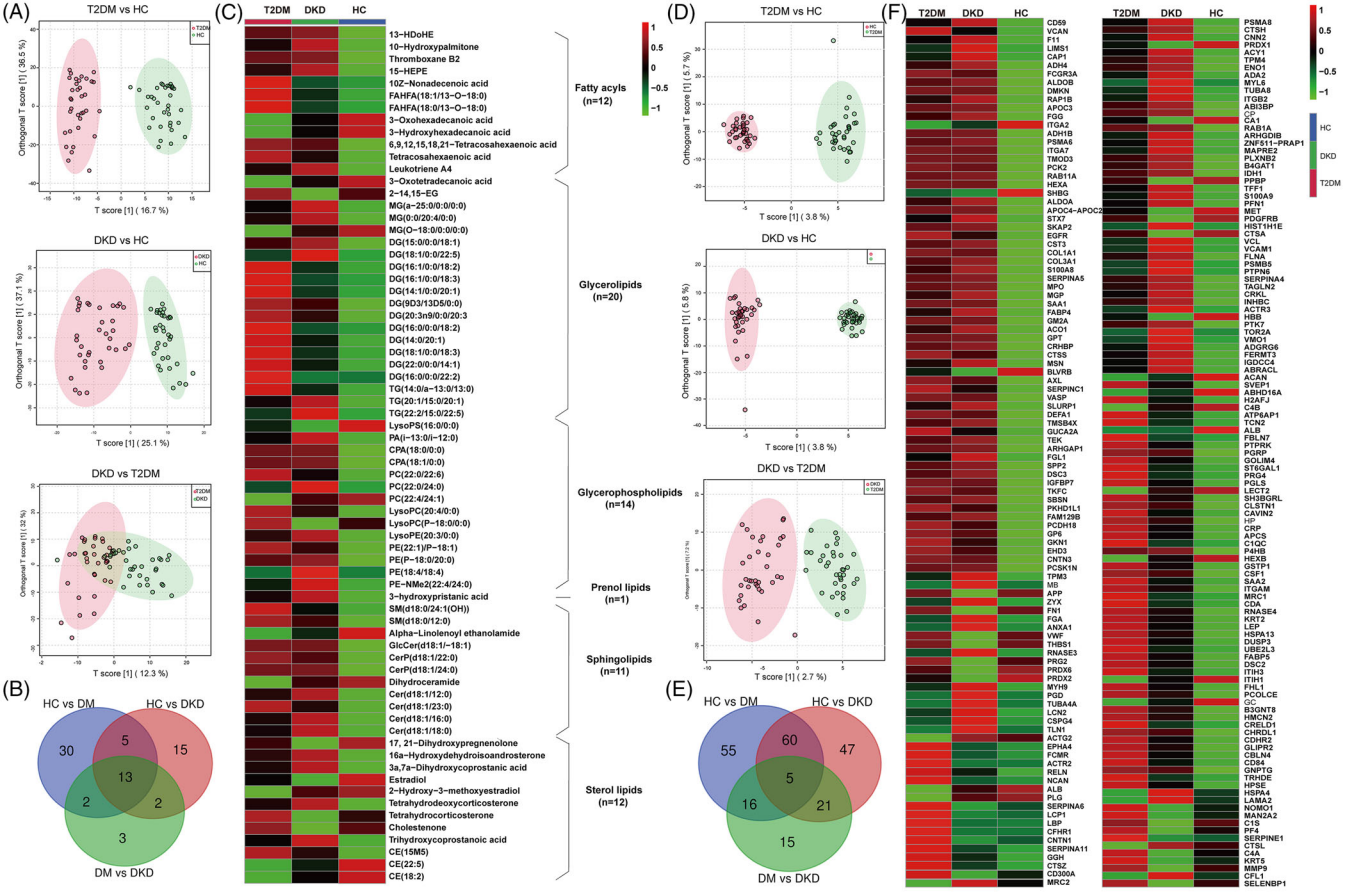
Lipidomics and proteomics results of discovery set. (A) Score plots of principal component analysis of each comparison group based on lipidomics modes from independent cohort 1. (B) Venn diagram of 70 differential lipids from three comparison groups in independent cohort 1. (C) Heatmap of 70 differential lipids in independent cohort 1. (D) Score plots of principal component analysis of each comparison group based on proteomics modes from independent cohort 1. (E) Venn diagram of 219 differential proteins from three comparison groups in independent cohort 1. (F) Heatmap of 219 differential proteins in independent cohort 1.

Proteomic data showed that protein content may vary depending on the physiological state of the individual (Figure 1D). With fold change (≥ 1.5 or ≤ .67) and *p* < .05 as screening criteria, 219 differential proteins were quantified (Figure S1B and Figure 1E–F), most of which were highly expressed in the both disease group (Table S3). In addition, the Gene Ontology and Kyoto Encyclopedia of Genes and Genomes analyses of the 219 proteins showed that complement and coagulation cascades, focal adhesions and phagosomes were significantly enriched, revealing that the development of DKD was related to pro‐inflammatory signals (Figures S1C and D).

Research is increasingly focusing on applying multi‐omics to identify ‘at‐risk’ profiles.^3^ At present, biomarkers for the risk of diabetes progressing to DKD at the single‐molecule level have been identified; however, their diagnostic efficacy is poor.^4^, ^5^ DKD is a complex secondary disease, and studies on risk markers at multiple molecular levels would be helpful in reflecting disease risk.^6^ We used support vector machine and convolutional neural network (CNN) models to evaluate the accuracy of single‐ or multi‐omics and found that the CNN model in multi‐omics showed significant advantages (Table S4), with the highest internal and prediction accuracies (100% and 90.48%, respectively). The neighborhood component analysis algorithm selected 58 fusion features (20%) from the 289 features, including 32 different proteins and 26 different lipids.

To reveal the intrinsic association of the 58 fusion features with DKD, Pearson correlation coefficient analysis was performed (Figure 2A). Twelve lipid metabolites showed significant association (*R* > .5) with 26 differentially expressed proteins (Figure 2B). By plotting the relative abundance of these lipid metabolites, we observed that the vast majority of lipids were significantly enriched in patients with T2DM than those with DKD (Figure 2C) and showed a linear increase with disease progression. A strong positive correlation between trihydroxycoprostanoic acid, Cer (d18:1/16:0), and 3α, 7 α‐dihydroxycoprostanic acid was observed (Figure 2D, *R* > .85, *p* < .01). These results suggest that DKD‐related proteins are associated with changes in serum lipid metabolite levels.

**FIGURE 2:**
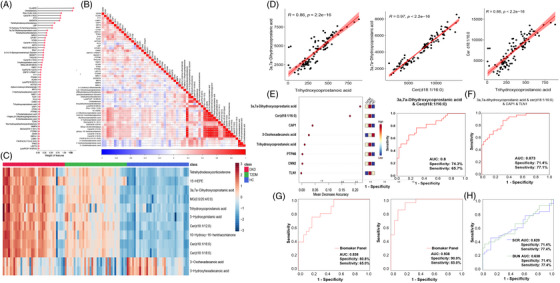
Determination and evaluation of diagnostic efficiency of biomarker panel. (A) Ranking of 58 fusion features in multiomics. (B) Pearson correlation of 58 fusion features. (C) Heatmap of 12 important differential lipid molecular features. (D) Pearson correlation analysis between 3a,7a‐dihydroxycoprostanic acid, cer(d18:1/16:0) and trihydroxycoprostanoic. (E) Mean decrease accuracy scores of eight key features. (F) Determination of biomarker panel by receiver operating characteristic (ROC) curve; (G) ROC curve analysis of independent cohort 2‐based biomarker panel (T2DM vs. DKD and HC vs. DKD); (H) ROC curve analysis of independent cohort 1‐based serum creatinine (SCR) and blood urea nitrogen (BUN).

In the test set, four lipid metabolites and four proteins in the 58 fusion features showed similar trends and content changes as that in the discovery set (Tables S5 and 6). Recently, several clinical histological studies have focused on the concept of “biomarker panel”.^2^, ^7^, ^8^ Based on the above results, we selected 3α, 7α‐dihydroxycoprostanic acid and Cer (d18:1/16:0) with an absolute high contribution to draw the receiver operating characteristic curve, with an area under the curve (AUC) of .800 (95% confidence interval [CI]: .698–.902), to establish the diagnostic distinction between T2DM and DKD (Figure 2E). Subsequently, the remaining six substances were added to obtain the best biomarker panel to predict the development of DKD, which was composed of 3α, 7α ‐dihydroxycoprostanic acid, Cer (d18:1/16:0), cyclase‐associated protein 1 (CAP1) and talin‐1 (TLN1) (AUC = .873; 95% CI: .794–.951) (Figure 2F and S2A–B).

We applied the obtained biomarker panel to the discovery (AUC = .838, 95% CI: .726–.950) and test sets (AUC = .938, 95% CI: .8670–1.000) that showed a strong diagnostic ability far higher than serum creatinine (SCR) (AUC = .620, 95% CI: .485–.755), and blood urea nitrogen (BUN) (AUC = .638, 95% CI: .506–.770) (Figure 2G and H). We found that the two lipid metabolites, Cer (d18:1/16:0) and 3α, 7α‐dihydroxycoprostanic acid, had prominent and robust positive correlations with hemoglobin A1c and glucose levels (Figures S2C). In addition, the positive correlations between CAP1, TLN1, SCR and BUN were stronger than those between the two lipid metabolites (Figures S2D). Furthermore, all four markers were positively correlated with a history of diabetes to varying degrees, with the two lipid metabolites being particularly significant (Figures S2E). This emphasizes the complementary nature and importance of a biomarker panel.

In conclusion, this study combined multiple bioinformatic tools and learning algorithms to synthetically identify the optimal diagnosis of a disease biomarker panel. Our findings provide insights for the integrated modelling of multi‐omics data and new research opportunities for T2DM complications. Furthermore, the combined use of two powerful histological techniques, lipidomics and proteomics, provided a comprehensive understanding of this disease.^9^, ^10^ The advent of DL will enable the handling of large amounts of high‐dimensional and complex‐structured data, further enabling the identification of key metabolic features.

## LIMITATIONS

This study used training models from small populations to validate large cohorts because of complications such as sample collection and time constraints, which may have resulted in some features being neglected. Therefore, in future studies, attention should be paid to the cohort settings (usually 8:1 to 4:1).

## CONFLICT OF INTEREST STATEMENT

The authors declare no conflict of interest.

## Supporting information

Supporting Information

Supporting Information

Supporting Information
