# Supplementary figures and images for: Deep learning‐based multi‐omics study reveals the polymolecular phenotypic of diabetic kidney disease
Source: Clin Transl Med. 2023 Jun 8;13(6):e1301. doi: 10.1002/ctm2.1301 (PMC10248822; doi:10.1002/ctm2.1301)

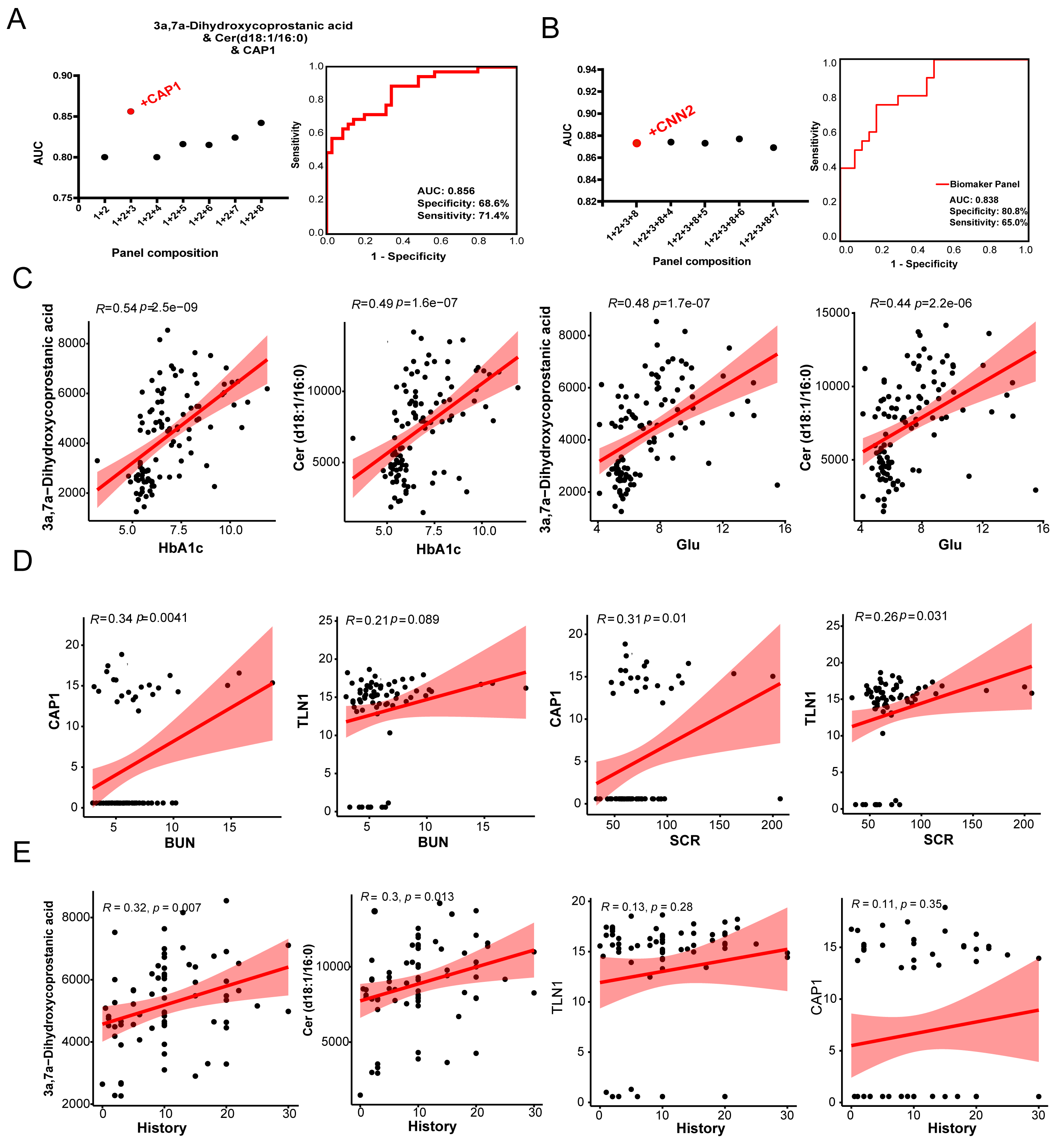

Supplement: Supplementary file 2 — Supporting Information [file CTM2-13-e1301-s002.tif]
