# Supplementary material for: Deep learning‐based multi‐omics study reveals the polymolecular phenotypic of diabetic kidney disease
Source: Clin Transl Med. 2023 Jun 8;13(6):e1301. doi: 10.1002/ctm2.1301 (PMC10248822; doi:10.1002/ctm2.1301)
Supplement: Supplementary file 3 — Supporting Information [file CTM2-13-e1301-s003.docx]

**Supplementary Materials**

**Subject criteria**

**Diagnostic Criteria**

In this study, the diagnostic criteria for type 2 diabetes (T2DM) refer to the 2019 World Health Organization (WHO) published diagnostic criteria for T2DM ^[1]^, which are fasting blood glucose level ≥ 7.0 mmol/L (126 mg/dL) and postprandial blood glucose ≥ 11.1 mmol/L (200 mg/dL).

The diagnostic criteria for diabetic kidney disease (DKD) refer to the American Diabetes Association^[2,3]^, which are fasting blood glucose level ≥ 7.0 mmol/L, urinary albumin/creatinine ratio (ACR) ≥ 30 mg/g for more than 3 months or estimated glomerular filtration rate (eGFR) < 60 mL/min/1.73m^2^.

**Inclusion Criteria**

The participants included in this study were those: 1) who met the relevant diagnostic criteria for DM or DKD and were diagnosed; 2) with duration of DM >1 year; 3) who aged between 18 and 80 years, regardless of gender; 4) with clear consciousness, no intellectual impairment, and ability to communicate normally; and 4) who participated voluntarily after being informed about this study.

**Exclusion Criteria**

The excluded patients were those: 1) with T2DM with FBG level <5.6 mmol/L, 2 h-PG level post-OGTT <7.8 mmol/L, and transient elevated blood glucose level due to other stress factors; 2) with primary kidney disease; 3) with severe cardiovascular, hepatic, or renal dysfunction; 4) with HIV infection, pregnancy, cancer, severe infections, or malignant tumor; 5) with severe mental illness and cognitive dysfunction; 6) who were diagnosed with type I diabetes or type I DKD; and 7) with acute complications of diabetes.

**Supplementary Table1.** Characteristics of the participants included in the Independent Cohort 2.

| **Trait** | **HC (n=100)** | **DM (n=100)** | **DKD (n=100)** |
| --- | --- | --- | --- |
| Age (years) | 61.13±6.17 | 61.24±5.07 | 61.10±7.43 |
| Sex (male/female) | 50/50 | 50/50 | 50/50 |
| BMI (kg/m2) | 25.08±4.08 | 26.35±3.1 | 25.54±3.1 |
| SBP (mmHg) | 116.95±8.6 | 127.20±14.15 | 134.03±13.53 |
| DBP (mmHg) | 72.95±10.11 | 74.0±7.9 | 80.19±8.76 |
| HbA1c (%) | 6.13±0.51 | 7.44±1.34 | 8.18±1.63 |
| eGFR (ml/min) | N/A | 93.89±13.82 | 94.72±16.09 |
| Glu(mmol/L) | 5.12±0.4 | 7.48±2.76 | 8.28±3.15 |
| SCR (μmol/L) | 72.66±13.77 | 76.57±14.81 | 88.47±18.68 |
| BUN (mmol/L) | 5.54±1.49 | 5.9±1.16 | 7.79±2.73 |
| TG (mmol/L) | 1.17±0.71 | 2.3±2.54 | 2.28±2.44 |
| TC (mmol/L) | 4.16±0.66 | 5.17±1.12 | 5.57±0.94 |
| HDL (mmol/L) | 1.37±0.21 | 1.18±0.42 | 1.21±0.30 |
| LDL (mmol/L) | 2.68±0.60 | 3.08±0.97 | 3.28±1.14 |

Data are mean ± SD for continuous measures and n for categorical measure. HC, healthy control group; DM, type 2 diabetes mellitus group; DKD, diabetic kidney disease group.

**Proteomics Analysis**

***Sample preparation***

The serum sample for data independent acquisition (DIA) analysis was denatured in 2% SDS buffer containing 50 mM DTT for 20 min at room temperature and then boiled at 100℃ for 5 min. The protein sample was alkylated for 1 h at room temperature in the dark by the addition of a final concentration of 200 mM iodoacetamide (IAA). Thereafter, five times volume of pre-cooled acetone was added, and the proteins precipitated overnight in the refrigerator at -20℃. Finally, the protein precipitates were resolved and digested by sequencing-grade modified trypsin (Promega) at a protein-to-enzyme ratio of 50:1 at 37℃ overnight. Tryptic peptides were collected by centrifugation at 14,000 *g* for 20 min at 20°C. The tryptic peptides were treated with 1% trifluoroacetic acid (TFA), purified using C_18_ Ziptips, and eluted with 0.1% TFA in 50%–70% acetonitrile. The eluted peptides were lyophilized using a SpeedVac (ThermoSavant) and resuspended in 1% formic acid 5% acetonitrile. The iRT peptides (Biognosys, Schlieren, Switzerland) were spiked into the sample prior to analysis according to manufacturer instructions.

***DIA sample acquisition***

To best resolve the DIA data based on the spectral library of the DDA data, the DIA MS/MS acquisition was performed with the same LC-MS systems and the same LC linear gradient method as DDA. For MS/MS acquisition, the DIA method was set 50 variable isolation windows according to the full width at half maximum, and the specific window lists were constructed based on the respective DDA data of the pooled sample. The full scan was set at a resolution of 1,200,000 over an m/z range of 350 to 1500, followed by DIA scans with a resolution of 30,000 (CE: 30% +/- 5, AGC target: 1e6, and maximal injection time: 54 ms).

***DIA data analysis***

The DIA raw files were analyzed in Spectronaut X (Biognosys, Schlieren, Switzerland). The default settings were used for targeted analysis of DIA data in Spectronaut. In brief, retention time prediction type was set to dynamic iRT and correction factor for window 1. Interference correction on MS2 level was enabled. Systematic variance was normalized using a local normalization strategy. The false discovery rate (FDR) was estimated with the mProphet approach and set to 1% at peptide precursor level and 1% at protein level. The protein intensity was determined by the intensity of their respective peptides that was calculated by the peak areas of their respective fragment ions of MS2. All results were filtered by a Q value cutoff of 0.01 (corresponds to a FDR of 1%).

**Lipidomics Analysis**

***Sample processing***

After serum thawing, 80 μL of serum was placed in a 1.5 mL centrifuge tube; 320 μL of solvent mixture (CH_2_CL_2_/methanol 3:1, v/v) was added, followed by mixing on a vortex for 60 s and then centrifugation at 13,000 rpm for 10 min. An aliquot of 80 μL of the organic phase layer (lower CH_2_CL_2_ layer) was isolated, dried with nitrogen at room temperature, and then dissolved in 160 μL of solvent mixture (acetonitrile/isopropanol 1:1, v/v), followed by centrifugation at 13,000 rpm for 10 min; thereafter, 150 μL of the supernatant was used for lipidomic analysis. A 20 μL aliquot of each sample was taken, vortexed, and mixed to prepare QC samples for methodological investigation.

***Instrument conditions***

In the positive ionization mode of the Waters Xevo G2-XS Q-TOF mass spectrometry system (Waters Corporation, USA), high-purity N_2_ was used as the auxiliary spray ionization and desolventizing gas, and the conditions were as follows: atomization gas pressure, 310 KPa; desolventizing gas flow rate, 800 L/h; cone hole backblast nitrogen flow rate, 50 L/h; desolventizing gas temperature, 400℃; capillary voltage, 3.0 kV; cone well voltage, 40 V; ionization source temperature, 120℃. The chromatographic column ACQUITY UPLC BEH C18 (2.1 × 100 mm, 1.7 μm) was used in a Waters ACQUITY UPLC system (Waters Corporation, USA), with the following conditions: injection volume, 2 μL; column temperature, 50 ℃; flow rate, 0.25 mL/min; mobile phase A, water:methanol:acetonitrile (3:1:1, v/v) with 5 mM/L ammonium formate; mobile phase B, isopropanol with 10 mM ammonium formate; gradient elution mode (0–0.5 min, 80%A; 0.5–5.0 min, 80%–60%A; 5.0–15.0 min, 60%–2%A; 15.0–17.0 min, 2%A; 17.0–17.1 min, 2%–80%; 17.1–20 min, 80%A).

***Data processing***

Multivariate statistical analysis and data integration analysis were used to discover differential metabolic biomarkers. The data analysis process was as follows: first, peak discovery, peak alignment, and peak filtering of raw data were performed using the MarkerLynx Application Manager version 4.1 (Waters, USA) data processing system to identify potential discriminant variables. Subsequently, the processed data were imported into SIMCA-P11.5 statistical software (Umetrics, Sweden) for unsupervised principal component analysis (PCA). Variables of variance were initially screened according to the variable importance in the projection >1 in the OPLS-DA model for metabolic ions. Subsequently, independent sample t-tests were performed using SPSS 17.0 software to determine whether the metabolites were significantly altered from a statistical perspective, and substances with P value <0.05 were selected as significantly different metabolites. The m/z values of significantly different metabolites were used to find possible substances in the HMDB (http://www.hmdb.ca/) database, Chemspider (http://www.chemspider.com/) database, as well as information from standards, MS/MS analysis, metabolite database, and literature to identify candidate biomarkers.

**Deep learning**

***CNN*** ***model***

The original data of proteomics and matched differential lipidomics characteristics of 105 samples constitute the data set of DL in this study. The original feature variables are screened by Neighbourhood component analysis, and the top 20% of features are selected. The samples are divided into two groups, including the training set (84 samples, 80%) and the test set (21 samples, 20%). The sample proportion of the three groups (HC, type 2 diabetes mellitus, DKD) in each group remained the same. CNN model framework is selected to realize the integration of multiomics data (MATLAB R 2021a). CNN model includes five parts: input layer, convolution layer, pooling layer, full connection layer, and output layer. The proteomics and lipidomics data of integrated analysis constitute the model input layer. The function of the convolution layer is to filter the features of the input layer data, and the output features will be transferred to the pooling layer for feature selection and information filtering. The function of the full connection layer is to make a nonlinear combination of the extracted features to obtain the output. Finally, the model category is defined by the clinical classification results. The training set is cross validated 10 times, and the model was tested on the test dataset. Continuous training and verification operations are performed during iterative learning to prevent model overfitting.

***SVM*** ***model***

Similarly, single-omics and multi-omics samples before and after feature extraction were used as analysis data, and an SVM classification and prediction model for DKD was established using MATLAB R 2021a software. Randomly divide the samples into a training set (2/3) and a testing set (1/3). The training set is used for computer learning and training, and the data from the testing set is used to determine the accuracy of the model. Using sigmoid as the kernel function for modeling, and obtaining the optimal parameters of the model through cross validation. Among them, -g is used to set the γ Parameter settings, default value is 1/k (k is the number of categories), -r is used to set coef0 in the kernel function, with a default value of 0.

**Statistical and Bioinformatics Analysis**

The clinical baseline matching of biochemical indexes of each subject selects one-way ANOVA or nonparametric test according to whether the data distribution conforms to the normal, to judge whether there are statistical differences among the indexes of each group. Independent sample t-test (approximate t-test) or nonparametric Mann-Whitney U test are selected to screen statistically significant potential differential metabolites and proteins between every two groups. Results were considered statistically significant at *p* < 0.05.

**References**

[1] Expert Committee on the Diagnosis and Classification of Diabetes Mellitus. Report of the Expert Committee on the Diagnosis and Classification of Diabetes Mellitus. Diabetes Care, 2003; 26(Suppl. 1):,S5-S20.

[2] Gross JL, de Azevedo MJ, Silveiro SP, Canani LH, Caramori ML, Zelmanovitz T. Diabetic nephropathy: diagnosis, prevention, and treatment. Diabetes Care. 2005; 28(1): 164-76.

[3] Foundation N K. KDOQI Clinical practice guidelines and clinical practice recommendations for diabetes and chronic kidney disease[J]. American Journal of Kidney Diseases. 2007; 49: S1-180.

**Supplementary Table 2. The70 metabolites information**

| **NO.** | **Ret.T** | **Molecular Formula** | **Metabolites** | **T2DM/HC** | | | **DKD/HC** | | | **T2DM/DKD** | | |
| --- | --- | --- | --- | --- | --- | --- | --- | --- | --- | --- | --- | --- |
|  |  |  |  | **Fold Change** | ***p*** | **FDR** | **Fold Change** | ***p*** | **FDR** | **Fold Change** | ***p*** | **FDR** |
| 1 | 10.11 | C_28_H_48_O_5_ | Trihydroxycoprostanoic acid | 1.69 | 2.38E-07 | 1.39E-06 | 2.39 | 1.94E-14 | 1.36E-13 | 0.71 | 4.77E-05 | 5.56E-04 |
| 2 | 7.76 | C_20_H_34_O_6_ | Thromboxane B2 | 1.94 | 1.63E-03 | 5.43E-03 | 2.01 | 6.58E-05 | 3.07E-04 | 0.97 | 0.85 | 0.90 |
| 3 | 14.47 | C_62_H_106_O_6_ | TG(22:2/15:0/22:5) | 1.12 | 0.52 | 0.55 | 1.41 | 4.35E-02 | 7.61E-02 | 0.80 | 0.16 | 0.28 |
| 4 | 14.40 | C_58_H_108_O_6_ | TG(20:1/15:0/20:1) | 1.21 | 0.23 | 0.27 | 1.36 | 5.39E-02 | 8.98E-02 | 0.89 | 0.44 | 0.56 |
| 5 | 13.99 | C_43_H_82_O_6_ | TG(14:0/a-13:0/13:0)[rac] | 1.33 | 3.99E-02 | 5.28E-02 | 1.12 | 0.33 | 0.36 | 1.19 | 0.20 | 0.33 |
| 6 | 6.91 | C_21_H_34_O_3_ | Tetrahydrodeoxycorticosterone | 1.96 | 5.97E-17 | 1.39E-15 | 2.56 | 1.06E-17 | 1.48E-16 | 0.77 | 8.54E-05 | 8.54E-04 |
| 7 | 7.19 | C_21_H_34_O_4_ | Tetrahydrocorticosterone | 1.07 | 0.43 | 0.48 | 0.85 | 9.95E-02 | 0.14 | 1.26 | 1.43E-02 | 6.24E-02 |
| 8 | 8.51 | C_24_H_36_O_2_ | Tetracosahexaenoic acid | 1.74 | 9.08E-03 | 1.77E-02 | 1.46 | 2.56E-02 | 5.11E-02 | 1.19 | 0.32 | 0.46 |
| 9 | 14.27 | C_47_H_93_N_2_O7_P_ | SM(d18:0/24:1(OH)) | 1.55 | 4.48E-03 | 1.16E-02 | 1.25 | 9.42E-02 | 0.14 | 1.24 | 0.14 | 0.25 |
| 10 | 14.71 | C_35_H_75_N_2_O_6_P | SM(d18:0/12:0) | 1.67 | 1.91E-02 | 2.96E-02 | 1.49 | 2.39E-02 | 5.07E-02 | 1.12 | 0.55 | 0.66 |
| 11 | 14.43 | C_53_H_98_NO_8_P | PE-NMe2(22:4/24:0) | 1.16 | 0.36 | 0.42 | 1.39 | 2.87E-02 | 5.43E-02 | 0.83 | 0.22 | 0.36 |
| 12 | 11.05 | C_43_H_86_NO_7_P | PE(P-18:0/20:0) | 1.52 | 2.85E-02 | 3.91E-02 | 1.61 | 5.02E-03 | 1.30E-02 | 0.94 | 0.72 | 0.77 |
| 13 | 10.97 | C_45_H_86_NO_7_P | PE(22:1)/P-18:1) | 1.41 | 1.58E-02 | 2.63E-02 | 1.28 | 8.12E-02 | 0.12 | 1.10 | 0.51 | 0.63 |
| 14 | 12.26 | C_41_H_66_NO_8_P | PE(18:4/18:4) | 1.01 | 0.89 | 0.89 | 1.26 | 2.16E-03 | 7.20E-03 | 0.80 | 9.46E-03 | 4.42E-02 |
| 15 | 14.67 | C_54_H_98_NO_8_P | PC(22:4/24:1) | 0.73 | 9.65E-03 | 1.78E-02 | 0.93 | 0.48 | 0.49 | 0.78 | 2.54E-02 | 8.46E-02 |
| 16 | 14.51 | C_54_H_108_NO_8_P | PC(22:0/24:0) | 1.09 | 0.56 | 0.58 | 1.36 | 2.26E-02 | 5.10E-02 | 0.80 | 9.13E-02 | 0.19 |
| 17 | 14.18 | C_52_H_92_NO_8_P | PC(22:0/22:6) | 1.84 | 1.65E-02 | 2.62E-02 | 1.48 | 4.23E-02 | 7.60E-02 | 1.24 | 0.32 | 0.46 |
| 18 | 9.30 | C_28_H_55_O_8_P | PA(i-13:0/i-12:0) | 1.94 | 6.46E-09 | 4.11E-08 | 2.80 | 8.14E-12 | 4.75E-11 | 0.69 | 7.95E-04 | 4.64E-03 |
| 19 | 14.68 | C_21_H_44_O_3_ | MG(O-18:0/0:0/0:0) | 0.86 | 8.13E-02 | 0.10 | 0.95 | 0.451 | 0.48 | 0.90 | 0.28 | 0.43 |
| 20 | 12.20 | C_28_H_56_O_4_ | MG(a-25:0/0:0/0:0)[rac] | 1.51 | 2.24E-05 | 1.21E-04 | 2.05 | 2.35E-14 | 1.50E-13 | 0.74 | 2.62E-04 | 1.84E-03 |
| 21 | 9.83 | C_23_H_38_O_4_ | MG(0:0/20:4/0:0)2-Arachidonylglycerol | 1.72 | 1.33E-12 | 1.17E-11 | 2.19 | 4.96E-17 | 5.79E-16 | 0.78 | 1.80E-04 | 1.40E-03 |
| 22 | 9.21 | C_22_H_44_NO_9_P | LysoPS(16:0/0:0) | 0.82 | 0.24 | 0.29 | 0.71 | 2.44E-02 | 5.02E-02 | 1.16 | 0.40 | 0.54 |
| 23 | 6.39 | C_25_H_46_NO_7_P | LysoPE(20:3(5Z,8Z,11Z)/0:0) | 1.26 | 0.10 | 0.13 | 1.40 | 1.62E-02 | 3.90E-02 | 0.90 | 0.43 | 0.55 |
| 24 | 7.84 | C_26_H_54_NO_6_P | LysoPC(P-18:0/0:0) | 1.09 | 0.54 | 0.56 | 0.79 | 0.11 | 0.14 | 1.39 | 3.58E-02 | 0.10 |
| 25 | 6.08 | C_28_H_50_NO_7_P | LysoPC(20:4/0:0) | 1.32 | 2.87E-02 | 3.87E-02 | 1.21 | 0.19 | 0.22 | 1.09 | 0.56 | 0.65 |
| 26 | 9.23 | C_20_H_30_O_3_ | Leukotriene A4 | 1.85 | 4.04E-09 | 2.83E-08 | 2.38 | 1.18E-09 | 6.38E-09 | 0.78 | 1.86E-02 | 7.25E-02 |
| 27 | 11.65 | C_42_H_79_NO_8_ | GlcCer(d18:1/-18:1) | 1.43 | 3.27E-03 | 9.55E-03 | 1.42 | 4.62E-03 | 1.24E-02 | 1.00 | 0.98 | 0.98 |
| 28 | 14.64 | C_36_H_68_O_4_ | FAHFA(18:1/13-O-18:0) | 1.82 | 4.92E-03 | 1.23E-02 | 1.29 | 0.14 | 0.16 | 1.41 | 7.54E-02 | 0.16 |
| 29 | 14.79 | C_36_H_70_O_4_ | FAHFA(18:0/13-O-18:0) | 1.79 | 1.20E-03 | 4.43E-03 | 1.22 | 0.10 | 0.14 | 1.47 | 2.24E-02 | 7.84E-02 |
| 30 | 5.60 | C_18_H_24_O_2_ | Estradiol | 0.86 | 0.18 | 0.27 | 0.73 | 3.11E-03 | 9.07E-03 | 1.18 | 0.11 | 0.21 |
| 31 | 3.55 | C_19_H_39_NO_3_ | Dihydroceramide | 0.67 | 2.75E-02 | 3.93E-02 | 0.90 | 0.57 | 0.59 | 0.74 | 0.13 | 0.24 |
| 32 | 12.61 | C_45_H_76_O_7_ | DG(9D3/13D5/0:0) | 1.54 | 2.25E-02 | 3.34E-02 | 1.39 | 2.84E-02 | 5.52E-02 | 1.11 | 0.57 | 0.65 |
| 33 | 13.26 | C_39_H_74_O_5_ | DG(22:0/0:0/14:1n5) | 1.89 | 1.76E-03 | 5.61E-03 | 1.37 | 5.79E-02 | 9.42E-02 | 1.38 | 8.04E-02 | 0.17 |
| 34 | 12.64 | C_43_H_72_O_5_ | DG(20:3n9/0:0/20:3n9) | 1.63 | 6.47E-03 | 1.37E-02 | 1.42 | 2.37E-02 | 5.19E-02 | 1.14 | 0.42 | 0.56 |
| 35 | 12.87 | C_39_H_68_O_5_ | DG(18:1n9/0:0/18:3n6) | 1.66 | 5.98E-03 | 1.35E-02 | 1.23 | 0.12 | 0.15 | 1.36 | 7.04E-02 | 0.16 |
| 36 | 12.27 | C_43_H_72_O_5_ | DG(18:1n7/0:0/22:5n6) | 1.11 | 0.44 | 0.48 | 1.43 | 3.81E-03 | 1.07E-02 | 0.78 | 4.94E-02 | 0.13 |
| 37 | 12.46 | C_37_H_64_O_5_ | DG(16:1n7/0:0/18:3n3) | 1.71 | 7.67E-03 | 1.53E-02 | 1.17 | 0.33 | 0.35 | 1.46 | 4.78E-02 | 0.13 |
| 38 | 12.45 | C_38_H_68_O_5_ | DG(16:1n7/0:0/18:2n6) | 1.76 | 8.84E-04 | 3.44E-03 | 1.25 | 4.91E-02 | 8.38E-02 | 1.41 | 3.19E-02 | 9.72E-02 |
| 39 | 13.27 | C_41_H_76_O_5_ | DG(16:0/0:0/22:2n6) | 1.52 | 2.84E-02 | 3.98E-02 | 1.00 | 0.99 | 0.99 | 1.52 | 1.97E-02 | 7.25E-02 |
| 40 | 12.75 | C_38_H_70_O_5_ | DG(16:0/0:0/18:2n6) | 2.10 | 6.79E-04 | 3.17E-03 | 1.23 | 0.12 | 0.15 | 1.71 | 8.41E-03 | 4.21E-02 |
| 41 | 11.36 | C_36_H_68_O_5_ | DG(15:0/0:0/18:1n7) | 1.31 | 0.12 | 0.15 | 1.43 | 1.85E-02 | 4.32E-02 | 0.92 | 0.63 | 0.70 |
| 42 | 12.59 | C_37_H_68_O_5_ | DG(14:1n5/0:0/20:1n9) | 1.51 | 6.06E-03 | 1.33E-02 | 1.15 | 0.19 | 0.22 | 1.31 | 6.72E-02 | 0.16 |
| 43 | 12.87 | C_37_H_70_O_5_ | DG(14:0/20:1/0:0) | 1.80 | 3.73E-03 | 1.05E-02 | 1.33 | 7.89E-02 | 0.13 | 1.35 | 0.11 | 0.21 |
| 44 | 7.52 | C_21_H_39_O_6_P | CPA(18:1(9Z)/0:0) | 1.85 | 1.99E-02 | 3.03E-02 | 1.89 | 1.21E-03 | 4.47E-03 | 0.98 | 0.93 | 0.97 |
| 45 | 7.95 | C_21_H_41_O_6_P | CPA(18:0/0:0) | 2.37 | 1.15E-02 | 2.06E-02 | 2.42 | 4.94E-04 | 1.92E-03 | 0.98 | 0.93 | 0.96 |
| 46 | 8.33 | C_27_H_44_O | Cholestenone | 1.05 | 0.47 | 0.50 | 0.74 | 1.79E-04 | 7.37E-04 | 1.42 | 2.06E-05 | 3.61E-04 |
| 47 | 12.27 | C_42_H_84_NO_6_P | CerP(d18:1/24:0) | 1.56 | 2.37E-02 | 3.46E-02 | 1.58 | 1.58E-02 | 3.95E-02 | 0.99 | 0.97 | 0.98 |
| 48 | 11.81 | C_40_H_80_NO_6_P | CerP(d18:1/22:0) | 2.95 | 4.87E-04 | 2.43E-03 | 2.58 | 1.56E-03 | 5.47E-03 | 1.14 | 0.55 | 0.66 |
| 49 | 13.45 | C_41_H_81_NO_3_ | Cer(d18:1/23:0) | 1.45 | 6.52E-03 | 1.34E-02 | 1.28 | 3.21E-02 | 5.91E-02 | 1.13 | 0.39 | 0.53 |
| 50 | 14.15 | C_36_H_71_NO_3_ | Cer(d18:1/18:0) | 1.78 | 2.68E-13 | 3.13E-12 | 2.35 | 3.15E-15 | 2.75E-14 | 0.76 | 1.05E-04 | 9.20E-04 |
| 51 | 13.85 | C_34_H_67_NO_3_ | Cer(d18:1/16:0) | 1.77 | 1.81E-13 | 2.54E-12 | 2.27 | 4.00E-15 | 3.11E-14 | 0.78 | 4.99E-04 | 3.18E-03 |
| 52 | 12.42 | C_30_H_59_NO_3_ | Cer(d18:1/12:0) | 1.71 | 3.07E-13 | 3.07E-12 | 2.12 | 8.56E-16 | 8.56E-15 | 0.81 | 8.29E-04 | 4.46E-03 |
| 53 | 14.70 | C_49_H_78_O_2_ | CE(22:5) | 0.66 | 1.61E-02 | 2.62E-02 | 0.80 | 0.13 | 0.16 | 0.82 | 0.33 | 0.46 |
| 54 | 14.84 | C_45_H_76_O_2_ | CE(18:2) | 0.80 | 2.42E-03 | 7.38E-03 | 0.90 | 7.92E-02 | 0.12 | 0.89 | 0.11 | 0.21 |
| 55 | 14.21 | C_52_H_88_O_3_ | CE(15M5) | 1.51 | 4.07E-02 | 5.28E-02 | 1.33 | 9.58E-02 | 0.14 | 1.14 | 0.49 | 0.61 |
| 56 | 3.44 | C_20_H_35_NO_2_ | Alpha-Linolenoyl ethanolamide | 0.65 | 5.21E-03 | 1.26E-02 | 0.78 | 0.10 | 0.14 | 0.83 | 0.29 | 0.44 |
| 57 | 8.51 | C_24_H_36_O_2_ | 6,9,12,15,18,21-Tetracosahexaenoic acid | 2.45 | 7.96E-04 | 3.28E-03 | 2.21 | 1.40E-04 | 6.14E-04 | 1.11 | 0.63 | 0.71 |
| 58 | 14.85 | C_14_H_26_O_3_ | 3-Oxotetradecanoic acid | 0.82 | 1.39E-03 | 4.86E-03 | 0.93 | 0.14 | 0.16 | 0.89 | 6.15E-02 | 0.15 |
| 59 | 14.83 | C_16_H_30_O_3_ | 3-Oxohexadecanoic acid | 0.86 | 1.57E-02 | 2.67E-02 | 0.93 | 0.21 | 0.23 | 0.92 | 0.22 | 0.36 |
| 60 | 11.35 | C_19_H_38_O_3_ | 3-hydroxypristanic acid | 1.59 | 1.55E-12 | 1.20E-11 | 2.01 | 8.96E-19 | 2.09E-17 | 0.79 | 6.53E-06 | 4.57E-04 |
| 61 | 14.84 | C_16_H_32_O_3_ | 3-Hydroxyhexadecanoic acid | 0.83 | 3.78E-03 | 1.02E-02 | 0.92 | 0.13 | 0.16 | 0.90 | 0.11 | 0.21 |
| 62 | 9.83 | C_27_H_46_O_4_ | 3a,7a-Dihydroxycoprostanic acid | 1.83 | 2.42E-17 | 8.45E-16 | 2.38 | 1.48E-20 | 1.04E-18 | 0.77 | 1.56E-05 | 3.64E-04 |
| 63 | 8.21 | C_19_H_26_O_3_ | 2-Hydroxyestradiol-3-methyl ether | 0.70 | 9.56E-03 | 1.81E-02 | 0.93 | 0.65 | 0.66 | 0.75 | 7.44E-02 | 0.17 |
| 64 | 7.12 | C_23_H_38_O_5_ | 2-(14,15-Epoxyeicosatrienoyl) Glycerol | 1.04 | 0.65 | 0.66 | 0.83 | 7.94E-02 | 0.12 | 1.26 | 1.83E-02 | 7.52E-02 |
| 65 | 6.68 | C_21_H_32_O_4_ | 17alpha,21-Dihydroxypregnenolone | 0.91 | 0.43 | 0.48 | 0.69 | 2.65E-03 | 8.07E-03 | 1.31 | 3.31E-02 | 9.64E-02 |
| 66 | 9.11 | C_19_H_28_O_3_ | 16a-Hydroxydehydroisoandrosterone | 1.42 | 1.36E-02 | 2.37E-02 | 1.65 | 2.64E-03 | 8.40E-03 | 0.86 | 0.32 | 0.47 |
| 67 | 9.78 | C_20_H_30_O_3_ | 15-HEPE | 1.68 | 2.55E-23 | 1.78E-21 | 2.04 | 5.32E-20 | 1.86E-18 | 0.82 | 1.47E-05 | 5.13E-04 |
| 68 | 7.75 | C_22_H_34_O | 13-HDoHE | 2.20 | 6.89E-04 | 3.01E-03 | 2.36 | 6.77E-06 | 3.38E-05 | 0.93 | 0.71 | 0.77 |
| 69 | 12.32 | C_19_H_36_O_2_ | 10Z-Nonadecenoic acid | 1.97 | 5.45E-03 | 1.27E-02 | 1.20 | 0.19 | 0.22 | 1.64 | 3.17E-02 | 0.10 |
| 70 | 13.16 | C_31_H_62_O_2_ | 10-Hydroxy-16-hentriacontanone | 1.74 | 3.35E-16 | 5.87E-15 | 2.25 | 1.21E-18 | 2.13E-17 | 0.77 | 3.45E-05 | 4.83E-04 |
| HC, healthy control group; T2DM, type 2 diabetes mellitus group; DKD, diabetic kidney disease group. | | | | | | | | | | | | |

**Supplementary Table 3. The 219 proteins information**

| **NO.** | **Genes** | **Protein** | **T2DM/HC** | | | **DKD/HC** | | | **T2DM/DKD** | | |
| --- | --- | --- | --- | --- | --- | --- | --- | --- | --- | --- | --- |
|  |  |  | **Fold Change** | ***p*** | **FDR** | **Fold Change** | ***p*** | **FDR** | **Fold Change** | ***p*** | **FDR** |
| 1 | HSPA4 | Heat shock 70 kDa protein 4 | 0.79 | 0.46 | 0.69 | 1.52 | 0.09 | 0.33 | 0.52 | 0.02 | 0.30 |
| 2 | TPM3 | Tropomyosin alpha-3 chain | 1.17 | 0.27 | 0.54 | 1.46 | 6.20E-04 | 0.02 | 0.80 | 0.02 | 0.33 |
| 3 | LAMA2 | Laminin subunit alpha-2 | 0.97 | 0.65 | 0.82 | 1.07 | 0.18 | 0.44 | 0.90 | 0.04 | 0.42 |
| 4 | PSMA8 | Proteasome endopeptidase complex | 2.83 | 0.17 | 0.42 | 4.92 | 0.03 | 0.16 | 0.57 | 0.33 | 0.73 |
| 5 | CTSH | Pro-cathepsin H | 1.80 | 0.13 | 0.37 | 2.19 | 0.03 | 0.18 | 0.82 | 0.52 | 0.84 |
| 6 | NOMO1 | Nodal modulator 1 | 1.26 | 0.19 | 0.45 | 0.82 | 0.36 | 0.61 | 1.53 | 0.03 | 0.38 |
| 7 | ACAN | Aggrecan core protein | 0.36 | 0.04 | 0.21 | 0.56 | 0.19 | 0.45 | 0.65 | 0.43 | 0.80 |
| 8 | CNN2 | Calponin-2 | 1.66 | 0.15 | 0.41 | 2.49 | 2.19E-03 | 0.04 | 0.67 | 0.11 | 0.59 |
| 9 | SVEP1 | Sushi, von Willebrand factor type A, EGF and pentraxin domain-containing protein 1 | 1.44 | 0.03 | 0.18 | 1.26 | 0.24 | 0.51 | 1.15 | 0.34 | 0.74 |
| 10 | PRDX1 | Peroxiredoxin-1 | 0.75 | 0.22 | 0.49 | 0.54 | 0.02 | 0.14 | 1.39 | 0.28 | 0.73 |
| 11 | MAN2A2 | Alpha-mannosidase 2x | 1.31 | 0.18 | 0.45 | 0.80 | 0.40 | 0.64 | 1.65 | 0.03 | 0.36 |
| 12 | ADH4 | Alcohol dehydrogenase 4 | 3.94 | 0.04 | 0.21 | 5.19 | 0.01 | 0.11 | 0.76 | 0.56 | 0.85 |
| 13 | ABHD16A | Phosphatidylserine lipase ABHD16A | 0.98 | 1.38E-05 | 1.62E-03 | 0.98 | 3.79E-03 | 0.05 | 0.99 | 0.22 | 0.69 |
| 14 | H2AFJ | Histone H2A | 3.94 | 0.01 | 0.10 | 2.32 | 0.15 | 0.43 | 1.70 | 0.23 | 0.70 |
| 15 | ACY1 | Aminoacylase-1 | 1.72 | 0.09 | 0.31 | 2.43 | 1.01E-03 | 0.03 | 0.71 | 0.12 | 0.62 |
| 16 | FCGR3A | Low affinity immunoglobulin gamma Fc region receptor III-A | 4.32 | 3.66E-03 | 0.06 | 3.84 | 0.01 | 0.08 | 1.12 | 0.73 | 0.90 |
| 17 | TPM4 | Tropomyosin alpha-4 chain | 1.55 | 0.11 | 0.35 | 1.86 | 0.02 | 0.12 | 0.83 | 0.40 | 0.79 |
| 18 | ENO1 | Alpha-enolase | 1.06 | 0.16 | 0.42 | 1.09 | 0.05 | 0.23 | 0.98 | 1.97E-03 | 0.14 |
| 19 | CD59 | CD59 glycoprotein | 1.55 | 0.03 | 0.17 | 1.97 | 3.04E-05 | 2.18E-03 | 0.78 | 0.04 | 0.42 |
| 20 | ALDOB | Fructose-bisphosphate aldolase B | 1.04 | 0.01 | 0.07 | 1.05 | 6.36E-04 | 0.02 | 0.99 | 0.62 | 0.87 |
| 21 | C4B | Complement C4-B | 0.97 | 2.50E-03 | 0.05 | 0.99 | 0.22 | 0.50 | 0.98 | 0.04 | 0.42 |
| 22 | DMKN | Dermokine | 2.58 | 0.02 | 0.14 | 2.56 | 0.02 | 0.14 | 1.01 | 0.98 | 1.00 |
| 23 | RAP1B | Ras-related protein Rap-1b | 1.76 | 0.03 | 0.17 | 2.20 | 9.39E-04 | 0.03 | 0.80 | 0.23 | 0.70 |
| 24 | ATP6AP1 | V-type proton ATPase subunit S1 | 3.48 | 0.03 | 0.18 | 1.97 | 0.24 | 0.52 | 1.76 | 0.25 | 0.71 |
| 25 | MB | Myoglobin | 0.99 | 0.99 | 0.99 | 3.49 | 0.03 | 0.17 | 0.28 | 0.03 | 0.37 |
| 26 | APOC3 | Apolipoprotein C-III | 1.02 | 8.31E-06 | 1.62E-03 | 1.03 | 5.41E-08 | 2.13E-05 | 1.00 | 0.73 | 0.90 |
| 27 | ADA2 | Adenosine deaminase 2 | 1.07 | 0.39 | 0.63 | 1.17 | 0.02 | 0.14 | 0.92 | 0.08 | 0.54 |
| 28 | TCN2 | Transcobalamin-2 | 1.35 | 0.04 | 0.19 | 1.12 | 0.51 | 0.73 | 1.21 | 0.15 | 0.65 |
| 29 | ALB | Serum albumin | 0.97 | 2.21E-03 | 0.05 | 0.98 | 0.01 | 0.07 | 1.00 | 0.77 | 0.92 |
| 30 | MYL6 | Myosin light polypeptide 6 | 1.15 | 0.80 | 0.90 | 2.46 | 0.05 | 0.23 | 0.47 | 0.08 | 0.55 |
| 31 | FBLN7 | Fibulin-7 | 3.65 | 0.04 | 0.21 | 1.65 | 0.32 | 0.57 | 2.21 | 0.17 | 0.65 |
| 32 | TUBA8 | Tubulin alpha-8 chain | 1.41 | 0.31 | 0.58 | 2.19 | 0.01 | 0.08 | 0.65 | 0.09 | 0.56 |
| 33 | FGG | Fibrinogen gamma chain | 2.03 | 1.75E-04 | 0.01 | 2.43 | 8.20E-06 | 7.17E-04 | 0.83 | 0.15 | 0.65 |
| 34 | ACTG2 | Actin | 0.78 | 0.01 | 0.10 | 1.05 | 0.32 | 0.57 | 0.74 | 1.47E-03 | 0.13 |
| 35 | ITGB2 | Integrin beta-2 | 1.59 | 0.27 | 0.54 | 2.43 | 0.02 | 0.12 | 0.65 | 0.18 | 0.66 |
| 36 | ABI3BP | Target of Nesh-SH3 | 1.03 | 0.05 | 0.21 | 1.04 | 0.01 | 0.11 | 0.99 | 0.56 | 0.85 |
| 37 | GC | Vitamin D-binding protein | 1.01 | 0.04 | 0.19 | 1.01 | 0.02 | 0.15 | 1.00 | 0.87 | 0.95 |
| 38 | ITGA2 | Integrin alpha-2 | 0.72 | 2.61E-03 | 0.05 | 0.81 | 0.02 | 0.15 | 0.89 | 0.41 | 0.79 |
| 39 | ADH1B | Alcohol dehydrogenase 1B | 3.35 | 0.01 | 0.10 | 3.46 | 0.01 | 0.10 | 0.97 | 0.93 | 0.98 |
| 40 | CA1 | Carbonic anhydrase 1 | 0.79 | 0.19 | 0.46 | 0.55 | 0.01 | 0.07 | 1.42 | 0.15 | 0.65 |
| 41 | RAB1A | Ras-related protein Rab-1A | 1.92 | 0.08 | 0.29 | 2.04 | 0.05 | 0.23 | 0.94 | 0.84 | 0.94 |
| 42 | VCAN | Versican core protein | 3.67 | 2.91E-05 | 2.54E-03 | 2.27 | 0.03 | 0.16 | 1.62 | 0.04 | 0.41 |
| 43 | APP | Amyloid-beta precursor protein | 1.00 | 0.98 | 0.99 | 0.90 | 0.02 | 0.14 | 1.11 | 0.02 | 0.33 |
| 44 | EPHA4 | Ephrin type-A receptor 4 | 1.47 | 0.02 | 0.14 | 1.08 | 0.70 | 0.84 | 1.36 | 0.05 | 0.43 |
| 45 | PTPRK | Receptor-type tyrosine-protein phosphatase kappa | 2.57 | 0.04 | 0.19 | 1.86 | 0.19 | 0.47 | 1.38 | 0.39 | 0.78 |
| 46 | FCMR | Fas apoptotic inhibitory molecule 3 | 1.81 | 0.01 | 0.11 | 1.14 | 0.66 | 0.83 | 1.59 | 0.05 | 0.42 |
| 47 | ACTR2 | Actin-related protein 2 | 3.56 | 0.03 | 0.17 | 1.01 | 0.99 | 1.00 | 3.54 | 0.03 | 0.38 |
| 48 | GOLIM4 | Golgi integral membrane protein 4 | 3.75 | 0.01 | 0.08 | 2.43 | 0.10 | 0.34 | 1.54 | 0.27 | 0.72 |
| 49 | PSMA6 | Proteasome subunit alpha type-6 | 3.00 | 0.01 | 0.09 | 3.29 | 2.61E-03 | 0.04 | 0.91 | 0.74 | 0.90 |
| 50 | ITGA7 | Integrin alpha-7 | 1.78 | 0.03 | 0.17 | 1.76 | 0.04 | 0.19 | 1.01 | 0.96 | 0.98 |
| 51 | ZYX | Zyxin | 1.94 | 0.17 | 0.42 | 4.59 | 1.34E-04 | 0.01 | 0.42 | 0.01 | 0.23 |
| 52 | FN1 | Fibronectin | 1.01 | 0.40 | 0.64 | 0.85 | 0.01 | 0.11 | 1.19 | 0.01 | 0.23 |
| 53 | ARHGDIB | Rho GDP-dissociation inhibitor 2 | 2.30 | 0.21 | 0.48 | 4.14 | 0.02 | 0.13 | 0.56 | 0.22 | 0.69 |
| 54 | TMOD3 | Tropomodulin-3 | 4.62 | 0.02 | 0.15 | 4.63 | 0.02 | 0.15 | 1.00 | 1.00 | 1.00 |
| 55 | PCK2 | Phosphoenolpyruvate carboxykinase [GTP] | 6.58 | 0.02 | 0.15 | 6.20 | 0.02 | 0.15 | 1.06 | 0.91 | 0.97 |
| 56 | RAB11A | Ras-related protein Rab-11A | 4.43 | 0.01 | 0.07 | 4.72 | 3.02E-03 | 0.04 | 0.94 | 0.85 | 0.94 |
| 57 | HEXA | Beta-hexosaminidase | 2.21 | 0.03 | 0.18 | 2.19 | 0.03 | 0.18 | 1.01 | 0.97 | 0.99 |
| 58 | ZNF511-PRAP1 | ZNF511-PRAP1 readthrough | 1.15 | 0.23 | 0.50 | 1.33 | 4.70E-03 | 0.06 | 0.87 | 0.07 | 0.53 |
| 59 | ST6GAL1 | Beta-galactoside alpha-2,6-sialyltransferase 1 | 2.20 | 1.13E-03 | 0.03 | 1.47 | 0.18 | 0.44 | 1.49 | 0.05 | 0.44 |
| 60 | SHBG | Sex hormone-binding globulin | 0.97 | 1.85E-03 | 0.04 | 0.96 | 1.08E-03 | 0.03 | 1.01 | 0.66 | 0.88 |
| 61 | ALDOA | Fructose-bisphosphate aldolase | 1.46 | 0.05 | 0.21 | 1.66 | 3.07E-03 | 0.04 | 0.88 | 0.31 | 0.73 |
| 62 | RELN | Reelin | 1.49 | 3.32E-03 | 0.06 | 1.20 | 0.27 | 0.56 | 1.24 | 0.04 | 0.42 |
| 63 | NCAN | Neurocan core protein | 2.95 | 2.80E-03 | 0.05 | 1.31 | 0.57 | 0.76 | 2.26 | 0.01 | 0.28 |
| 64 | MAPRE2 | Microtubule-associated protein RP/EB family member 2 | 2.24 | 0.13 | 0.38 | 3.93 | 3.02E-03 | 0.04 | 0.57 | 0.12 | 0.62 |
| 65 | PGLS | 6-phosphogluconolactonase | 3.75 | 0.04 | 0.21 | 2.30 | 0.16 | 0.43 | 1.63 | 0.37 | 0.76 |
| 66 | LECT2 | Leukocyte cell-derived chemotaxin-2 | 0.68 | 0.01 | 0.11 | 0.89 | 0.36 | 0.61 | 0.76 | 0.13 | 0.62 |
| 67 | PLXNB2 | Plexin-B2 | 1.18 | 0.10 | 0.33 | 1.25 | 0.01 | 0.11 | 0.94 | 0.31 | 0.73 |
| 68 | STX7 | Syntaxin-7 | 3.43 | 0.04 | 0.21 | 5.51 | 0.01 | 0.08 | 0.62 | 0.29 | 0.73 |
| 69 | B4GAT1 | Beta-1,4-glucuronyltransferase 1 | 1.11 | 0.20 | 0.46 | 1.15 | 0.05 | 0.23 | 0.96 | 0.40 | 0.79 |
| 70 | SH3BGRL | SH3 domain-binding glutamic acid-rich-like protein | 2.53 | 0.04 | 0.21 | 1.90 | 0.19 | 0.47 | 1.33 | 0.47 | 0.80 |
| 71 | SKAP2 | Src kinase-associated phosphoprotein 2 | 7.04 | 3.24E-03 | 0.06 | 7.74 | 1.68E-03 | 0.03 | 0.91 | 0.80 | 0.93 |
| 72 | PGRP | Peptidoglycan recognition protein 1 | 1.24 | 0.18 | 0.45 | 1.15 | 0.41 | 0.65 | 1.07 | 0.62 | 0.87 |
| 73 | IDH1 | Isocitrate dehydrogenase [NADP] cytoplasmic | 1.30 | 0.08 | 0.29 | 1.39 | 0.02 | 0.15 | 0.94 | 0.56 | 0.85 |
| 74 | CLSTN1 | Calsyntenin-1 | 1.92 | 0.03 | 0.17 | 1.68 | 0.09 | 0.33 | 1.14 | 0.59 | 0.86 |
| 75 | CAVIN2 | Caveolae-associated protein 2 | 4.98 | 0.01 | 0.10 | 2.81 | 0.08 | 0.31 | 1.77 | 0.24 | 0.70 |
| 76 | EGFR | Epidermal growth factor receptor | 4.62 | 1.50E-04 | 0.01 | 3.43 | 0.01 | 0.08 | 1.35 | 0.28 | 0.73 |
| 77 | HP | Haptoglobin | 1.04 | 0.01 | 0.09 | 1.03 | 0.12 | 0.38 | 1.01 | 0.41 | 0.79 |
| 78 | PLG | Plasminogen | 1.00 | 0.33 | 0.58 | 1.00 | 0.56 | 0.76 | 1.00 | 0.68 | 0.89 |
| 79 | SERPINC1 | Antithrombin-III | 1.00 | 0.87 | 0.94 | 1.01 | 0.31 | 0.57 | 0.99 | 0.25 | 0.71 |
| 80 | CST3 | Cystatin-C | 1.03 | 1.05E-03 | 0.03 | 1.04 | 2.81E-04 | 0.01 | 0.99 | 0.55 | 0.85 |
| 81 | COL1A1 | Collagen alpha-1(I) chain | 1.42 | 3.92E-03 | 0.06 | 1.39 | 0.01 | 0.09 | 1.02 | 0.72 | 0.89 |
| 82 | COL3A1 | Collagen alpha-1(III) chain | 1.55 | 0.01 | 0.11 | 1.67 | 1.92E-03 | 0.03 | 0.93 | 0.52 | 0.84 |
| 83 | FGA | Fibrinogen alpha chain | 1.02 | 0.01 | 0.08 | 1.08 | 1.02E-03 | 0.03 | 0.94 | 0.01 | 0.23 |
| 84 | FGB | Fibrinogen beta chain | 1.03 | 0.45 | 0.68 | 1.22 | 1.39E-04 | 0.01 | 0.84 | 1.17E-03 | 0.12 |
| 85 | CRP | C-reactive protein | 4.27 | 0.01 | 0.07 | 2.81 | 0.07 | 0.29 | 1.52 | 0.29 | 0.73 |
| 86 | APCS | Serum amyloid P-component | 1.98 | 0.03 | 0.18 | 1.52 | 0.23 | 0.51 | 1.30 | 0.34 | 0.74 |
| 87 | C1QC | Complement C1q subcomponent subunit C | 1.03 | 1.39E-04 | 0.01 | 1.01 | 0.33 | 0.57 | 1.02 | 0.02 | 0.30 |
| 88 | PPBP | Platelet basic protein | 0.99 | 0.16 | 0.42 | 0.97 | 1.80E-03 | 0.03 | 1.02 | 0.02 | 0.29 |
| 89 | PF4 | Platelet factor 4 | 1.01 | 0.13 | 0.37 | 0.98 | 0.20 | 0.47 | 1.03 | 0.03 | 0.38 |
| 90 | F11 | Coagulation factor XI | 1.04 | 1.65E-05 | 1.62E-03 | 1.07 | 6.51E-13 | 5.13E-10 | 0.97 | 1.36E-04 | 0.05 |
| 91 | ANXA1 | Annexin A1 | 1.58 | 0.32 | 0.58 | 6.08 | 3.03E-03 | 0.04 | 0.26 | 0.01 | 0.26 |
| 92 | TFF1 | Trefoil factor 1 | 1.72 | 0.16 | 0.42 | 2.40 | 0.01 | 0.09 | 0.72 | 0.24 | 0.70 |
| 93 | VWF | von Willebrand factor | 1.00 | 0.62 | 0.80 | 0.96 | 0.02 | 0.14 | 1.05 | 0.01 | 0.27 |
| 94 | S100A8 | Protein S100-A8 | 3.93 | 1.22E-04 | 0.01 | 5.10 | 2.87E-07 | 7.54E-05 | 0.77 | 0.18 | 0.66 |
| 95 | SERPINE1 | Plasminogen activator inhibitor 1 | 2.48 | 0.10 | 0.32 | 0.74 | 0.60 | 0.78 | 3.34 | 0.04 | 0.42 |
| 96 | SERPINA5 | Plasma serine protease inhibitor | 1.42 | 0.01 | 0.10 | 1.47 | 3.11E-03 | 0.04 | 0.96 | 0.66 | 0.87 |
| 97 | MPO | Myeloperoxidase | 1.21 | 0.04 | 0.20 | 1.23 | 0.03 | 0.16 | 0.98 | 0.79 | 0.93 |
| 98 | S100A9 | Protein S100-A9 | 1.50 | 0.23 | 0.51 | 2.12 | 0.01 | 0.11 | 0.71 | 0.19 | 0.66 |
| 99 | P4HB | Protein disulfide-isomerase | 1.13 | 0.04 | 0.21 | 1.14 | 0.05 | 0.24 | 0.99 | 0.78 | 0.92 |
| 100 | HEXB | Beta-hexosaminidase subunit beta | 0.78 | 0.03 | 0.16 | 0.91 | 0.28 | 0.56 | 0.86 | 0.24 | 0.70 |
| 101 | CTSL | Cathepsin L1 | 0.83 | 0.13 | 0.38 | 1.07 | 0.44 | 0.68 | 0.78 | 0.03 | 0.36 |
| 102 | PFN1 | Profilin-1 | 1.03 | 3.22E-03 | 0.06 | 1.05 | 2.00E-06 | 2.26E-04 | 0.98 | 0.05 | 0.42 |
| 103 | THBS1 | Thrombospondin-1 | 1.00 | 0.78 | 0.90 | 0.97 | 0.01 | 0.07 | 1.03 | 4.48E-03 | 0.19 |
| 104 | SERPINA6 | Corticosteroid-binding globulin | 1.31 | 0.04 | 0.21 | 0.91 | 0.60 | 0.78 | 1.44 | 0.01 | 0.26 |
| 105 | MGP | Matrix Gla protein | 1.29 | 0.04 | 0.19 | 1.42 | 1.53E-03 | 0.03 | 0.91 | 0.19 | 0.66 |
| 106 | MET | Hepatocyte growth factor receptor | 0.75 | 0.13 | 0.37 | 0.49 | 1.48E-03 | 0.03 | 1.52 | 0.11 | 0.59 |
| 107 | GSTP1 | Glutathione S-transferase P | 1.36 | 0.01 | 0.07 | 1.21 | 0.17 | 0.44 | 1.13 | 0.18 | 0.66 |
| 108 | CSF1 | Macrophage colony-stimulating factor 1 | 1.63 | 0.05 | 0.21 | 1.48 | 0.12 | 0.38 | 1.10 | 0.65 | 0.87 |
| 109 | PDGFRB | Platelet-derived growth factor receptor beta | 0.86 | 0.62 | 0.80 | 0.48 | 0.05 | 0.23 | 1.80 | 0.15 | 0.65 |
| 110 | SAA1 | Serum amyloid A-1 protein | 1.06 | 2.83E-03 | 0.05 | 1.06 | 9.05E-04 | 0.03 | 1.00 | 1.00 | 1.00 |
| 111 | SAA2 | Serum amyloid A-2 protein | 1.20 | 2.50E-03 | 0.05 | 1.09 | 0.29 | 0.57 | 1.10 | 0.11 | 0.60 |
| 112 | HIST1H1E | Histone H1.4 | 1.69 | 0.31 | 0.58 | 4.74 | 0.02 | 0.14 | 0.36 | 0.07 | 0.54 |
| 113 | CTSA | Lysosomal protective protein | 0.95 | 0.26 | 0.53 | 0.82 | 0.01 | 0.08 | 1.16 | 0.09 | 0.56 |
| 114 | ITGAM | Integrin alpha-M | 1.39 | 0.03 | 0.18 | 1.23 | 0.24 | 0.51 | 1.13 | 0.37 | 0.76 |
| 115 | RNASE3 | Eosinophil cationic protein | 2.31 | 0.16 | 0.42 | 7.21 | 1.67E-03 | 0.03 | 0.32 | 0.02 | 0.32 |
| 116 | KRT5 | Keratin, type II cytoskeletal 5 | 1.15 | 0.11 | 0.35 | 0.91 | 0.46 | 0.70 | 1.26 | 0.03 | 0.36 |
| 117 | PRG2 | Bone marrow proteoglycan | 1.11 | 0.60 | 0.80 | 0.57 | 0.03 | 0.18 | 1.94 | 0.01 | 0.23 |
| 118 | LCP1 | Plastin-2 | 1.24 | 0.01 | 0.10 | 0.98 | 0.86 | 0.93 | 1.27 | 0.01 | 0.22 |
| 119 | MMP9 | Matrix metalloproteinase-9 | 1.07 | 0.27 | 0.54 | 0.83 | 0.07 | 0.29 | 1.30 | 0.01 | 0.22 |
| 120 | FABP4 | Fatty acid-binding protein, adipocyte | 1.68 | 0.02 | 0.15 | 2.08 | 2.14E-04 | 0.01 | 0.81 | 0.16 | 0.65 |
| 121 | GM2A | Ganglioside GM2 activator | 2.06 | 0.02 | 0.14 | 2.06 | 0.02 | 0.13 | 1.00 | 0.99 | 1.00 |
| 122 | VCL | Vinculin | 1.34 | 0.39 | 0.63 | 2.04 | 0.01 | 0.11 | 0.66 | 0.11 | 0.59 |
| 123 | LBP | Lipopolysaccharide-binding protein | 1.04 | 4.58E-04 | 0.02 | 1.00 | 0.85 | 0.92 | 1.04 | 2.76E-03 | 0.15 |
| 124 | VCAM1 | Vascular cell adhesion protein 1 | 1.01 | 0.49 | 0.71 | 1.04 | 0.03 | 0.17 | 0.97 | 0.12 | 0.62 |
| 125 | ITIH1 | Inter-alpha-trypsin inhibitor heavy chain H1 | 1.00 | 0.78 | 0.90 | 0.99 | 0.29 | 0.57 | 1.01 | 0.19 | 0.66 |
| 126 | FLNA | Filamin-A | 1.30 | 0.08 | 0.29 | 1.47 | 2.94E-03 | 0.04 | 0.88 | 0.20 | 0.68 |
| 127 | ACO1 | Cytoplasmic aconitate hydratase | 4.26 | 0.01 | 0.10 | 5.79 | 3.62E-03 | 0.05 | 0.74 | 0.44 | 0.80 |
| 128 | MRC1 | Macrophage mannose receptor 1 | 1.04 | 6.06E-04 | 0.02 | 1.01 | 0.29 | 0.57 | 1.02 | 2.65E-03 | 0.15 |
| 129 | CFL1 | Cofilin-1 | 0.99 | 0.29 | 0.56 | 1.03 | 0.01 | 0.09 | 0.96 | 3.68E-03 | 0.17 |
| 130 | GPT | Alanine aminotransferase 1 | 2.28 | 0.02 | 0.14 | 2.32 | 0.02 | 0.12 | 0.98 | 0.95 | 0.98 |
| 131 | CRHBP | Corticotropin-releasing factor-binding protein | 2.20 | 0.01 | 0.07 | 2.38 | 1.99E-03 | 0.03 | 0.92 | 0.70 | 0.89 |
| 132 | CTSS | Cathepsin S | 1.99 | 1.40E-03 | 0.04 | 1.76 | 0.02 | 0.12 | 1.13 | 0.45 | 0.80 |
| 133 | MSN | Moesin | 1.37 | 0.04 | 0.19 | 1.61 | 2.59E-04 | 0.01 | 0.85 | 0.09 | 0.56 |
| 134 | PSMB5 | Proteasome subunit beta type-5 | 1.72 | 0.33 | 0.58 | 3.73 | 0.05 | 0.23 | 0.46 | 0.19 | 0.66 |
| 135 | PTPN6 | Tyrosine-protein phosphatase non-receptor type 6 | 1.93 | 0.31 | 0.58 | 4.67 | 0.02 | 0.14 | 0.41 | 0.13 | 0.62 |
| 136 | SERPINA4 | Kallistatin | 1.36 | 0.19 | 0.45 | 1.53 | 0.05 | 0.23 | 0.89 | 0.52 | 0.84 |
| 137 | PRDX6 | Peroxiredoxin-6 | 0.85 | 0.66 | 0.82 | 0.26 | 0.01 | 0.08 | 3.24 | 0.02 | 0.33 |
| 138 | BLVRB | Flavin reductase (NADPH) | 0.76 | 0.04 | 0.19 | 0.59 | 7.09E-04 | 0.02 | 1.29 | 0.20 | 0.66 |
| 139 | AXL | Tyrosine-protein kinase receptor UFO | 5.27 | 0.01 | 0.07 | 4.70 | 0.01 | 0.11 | 1.12 | 0.78 | 0.92 |
| 140 | PRDX2 | Peroxiredoxin-2 | 0.98 | 0.08 | 0.29 | 0.94 | 9.74E-05 | 0.01 | 1.04 | 0.01 | 0.27 |
| 141 | CDA | Cytidine deaminase | 3.19 | 4.54E-03 | 0.07 | 1.72 | 0.24 | 0.51 | 1.85 | 0.07 | 0.53 |
| 142 | RNASE4 | Ribonuclease 4 | 1.15 | 0.03 | 0.18 | 1.09 | 0.29 | 0.57 | 1.05 | 0.31 | 0.73 |
| 143 | MYH9 | Myosin-9 | 1.56 | 0.24 | 0.51 | 2.67 | 1.63E-03 | 0.03 | 0.58 | 0.05 | 0.42 |
| 144 | KRT2 | Keratin, type II cytoskeletal 2 epidermal | 2.45 | 0.01 | 0.07 | 1.82 | 0.09 | 0.31 | 1.34 | 0.23 | 0.70 |
| 145 | TAGLN2 | Transgelin-2;Transgelin-2 (Fragment) | 2.69 | 0.06 | 0.25 | 3.64 | 0.01 | 0.08 | 0.74 | 0.42 | 0.79 |
| 146 | LEP | Leptin | 2.73 | 0.02 | 0.14 | 1.91 | 0.17 | 0.43 | 1.43 | 0.32 | 0.73 |
| 147 | CRKL | Crk-like protein | 2.45 | 0.10 | 0.33 | 3.70 | 0.01 | 0.11 | 0.66 | 0.32 | 0.73 |
| 148 | LIMS1 | LIM and senescent cell antigen-like-containing domain protein 1 | 3.13 | 0.05 | 0.21 | 7.53 | 2.26E-05 | 1.78E-03 | 0.42 | 0.01 | 0.23 |
| 149 | HSPA13 | Heat shock 70 kDa protein 13 | 1.70 | 0.05 | 0.21 | 1.43 | 0.22 | 0.50 | 1.19 | 0.47 | 0.80 |
| 150 | VASP | Vasodilator-stimulated phosphoprotein | 4.78 | 0.01 | 0.07 | 4.51 | 0.01 | 0.08 | 1.06 | 0.87 | 0.95 |
| 151 | DUSP3 | Dual specificity protein phosphatase 3 | 3.40 | 0.03 | 0.17 | 2.21 | 0.17 | 0.44 | 1.54 | 0.34 | 0.74 |
| 152 | PGD | 6-phosphogluconate dehydrogenase, decarboxylating | 1.08 | 0.89 | 0.95 | 3.62 | 0.02 | 0.12 | 0.30 | 0.02 | 0.33 |
| 153 | SLURP1 | Secreted Ly-6/uPAR-related protein 1 | 3.29 | 4.80E-03 | 0.07 | 4.69 | 4.37E-05 | 2.86E-03 | 0.70 | 0.17 | 0.65 |
| 154 | INHBC | Inhibin beta C chain | 1.02 | 4.61E-04 | 0.02 | 1.03 | 1.27E-06 | 2.00E-04 | 0.99 | 0.06 | 0.51 |
| 155 | DEFA1 | Neutrophil defensin 1;Neutrophil defensin 3 | 1.34 | 1.35E-03 | 0.04 | 1.29 | 0.01 | 0.08 | 1.04 | 0.28 | 0.73 |
| 156 | ACTR3 | Actin-related protein 3 | 2.17 | 0.17 | 0.42 | 5.14 | 0.01 | 0.11 | 0.42 | 0.10 | 0.57 |
| 157 | TMSB4X | Thymosin beta-4 | 1.75 | 2.67E-04 | 0.01 | 1.95 | 2.84E-06 | 2.79E-04 | 0.90 | 0.23 | 0.70 |
| 158 | UBE2L3 | Ubiquitin-conjugating enzyme E2 L3 | 3.16 | 0.01 | 0.11 | 1.85 | 0.23 | 0.51 | 1.71 | 0.18 | 0.66 |
| 159 | TUBA4A | Tubulin alpha-4A chain | 1.00 | 0.43 | 0.66 | 4.03 | 0.04 | 0.22 | 0.25 | 0.04 | 0.42 |
| 160 | HBB | Hemoglobin subunit beta | 0.97 | 0.07 | 0.27 | 0.95 | 0.01 | 0.08 | 1.02 | 0.09 | 0.56 |
| 161 | LCN2 | Neutrophil gelatinase-associated lipocalin | 1.24 | 0.38 | 0.62 | 1.79 | 2.56E-03 | 0.04 | 0.69 | 0.04 | 0.38 |
| 162 | FABP5 | Fatty acid-binding protein 5 | 3.39 | 0.03 | 0.17 | 2.35 | 0.17 | 0.43 | 1.44 | 0.42 | 0.79 |
| 163 | CAP1 | Adenylyl cyclase-associated protein 1 | 3.33 | 0.02 | 0.11 | 6.88 | 1.04E-06 | 2.00E-04 | 0.48 | 0.01 | 0.23 |
| 164 | DSC2 | Desmocollin-2 | 1.59 | 0.02 | 0.14 | 1.34 | 0.18 | 0.45 | 1.19 | 0.30 | 0.73 |
| 165 | GUCA2A | Guanylin | 4.76 | 2.51E-04 | 0.01 | 3.13 | 0.02 | 0.14 | 1.52 | 0.17 | 0.66 |
| 166 | TEK | Angiopoietin-1 receptor | 2.92 | 0.05 | 0.21 | 3.38 | 0.03 | 0.15 | 0.86 | 0.72 | 0.89 |
| 167 | CFHR1 | Complement factor H-related protein 1 | 1.03 | 4.05E-06 | 1.47E-03 | 1.00 | 1.00 | 1.00 | 1.03 | 4.31E-04 | 0.11 |
| 168 | ITIH3 | Inter-alpha-trypsin inhibitor heavy chain H3 | 1.03 | 5.08E-04 | 0.02 | 1.01 | 0.14 | 0.41 | 1.02 | 0.05 | 0.42 |
| 169 | ARHGAP1 | Rho GTPase-activating protein 1 | 3.89 | 0.01 | 0.10 | 4.14 | 0.01 | 0.07 | 0.94 | 0.86 | 0.95 |
| 170 | FGL1 | Fibrinogen-like protein 1 | 2.58 | 0.04 | 0.19 | 4.33 | 3.89E-04 | 0.01 | 0.60 | 0.09 | 0.56 |
| 171 | CNTN1 | Contactin-1 | 1.69 | 0.03 | 0.18 | 0.86 | 0.63 | 0.80 | 1.97 | 0.01 | 0.23 |
| 172 | SPP2 | Secreted phosphoprotein 24 | 1.03 | 4.81E-03 | 0.07 | 1.04 | 8.93E-04 | 0.03 | 1.00 | 0.61 | 0.86 |
| 173 | SELENBP1 | Methanethiol oxidase | 1.11 | 0.51 | 0.72 | 0.76 | 0.17 | 0.43 | 1.47 | 0.03 | 0.38 |
| 174 | PTK7 | Inactive tyrosine-protein kinase 7 | 1.77 | 0.08 | 0.28 | 2.07 | 0.02 | 0.14 | 0.86 | 0.56 | 0.85 |
| 175 | FHL1 | Four and a half LIM domains protein 1 | 3.03 | 0.02 | 0.15 | 2.12 | 0.14 | 0.41 | 1.43 | 0.36 | 0.75 |
| 176 | DSC3 | Desmocollin-3 | 1.28 | 0.03 | 0.18 | 1.35 | 0.01 | 0.08 | 0.95 | 0.53 | 0.84 |
| 177 | PCOLCE | Procollagen C-endopeptidase enhancer 1 | 1.03 | 0.01 | 0.07 | 1.02 | 0.04 | 0.21 | 1.01 | 0.34 | 0.74 |
| 178 | IGFBP7 | Insulin-like growth factor-binding protein 7 | 1.15 | 0.01 | 0.08 | 1.14 | 0.03 | 0.17 | 1.01 | 0.84 | 0.94 |
| 179 | TKFC | Triokinase/FMN cyclase | 2.37 | 0.01 | 0.07 | 2.17 | 0.02 | 0.14 | 1.09 | 0.71 | 0.89 |
| 180 | TOR2A | Torsin-2A | 1.18 | 0.73 | 0.87 | 2.50 | 0.03 | 0.17 | 0.47 | 0.06 | 0.51 |
| 181 | CSPG4 | Chondroitin sulfate proteoglycan 4 | 1.42 | 0.48 | 0.71 | 3.24 | 0.01 | 0.08 | 0.44 | 0.03 | 0.38 |
| 182 | SBSN | Suprabasin | 2.01 | 0.05 | 0.21 | 2.22 | 0.01 | 0.09 | 0.91 | 0.70 | 0.89 |
| 183 | VMO1 | Vitelline membrane outer layer protein 1 homolog | 1.12 | 0.61 | 0.80 | 1.50 | 0.05 | 0.23 | 0.75 | 0.14 | 0.65 |
| 184 | B3GNT8 | UDP-GlcNAc:betaGal beta-1,3-N-acetylglucosaminyltransferase 8 | 1.95 | 0.01 | 0.07 | 1.61 | 0.08 | 0.31 | 1.21 | 0.34 | 0.74 |
| 185 | ADGRG6 | Adhesion G-protein coupled receptor G6 | 1.18 | 0.30 | 0.57 | 1.40 | 0.01 | 0.08 | 0.84 | 0.10 | 0.57 |
| 186 | SERPINA11 | Serpin A11 | 1.53 | 1.58E-05 | 1.62E-03 | 1.13 | 0.38 | 0.62 | 1.35 | 3.29E-03 | 0.16 |
| 187 | FERMT3 | Fermitin family homolog 3 | 1.57 | 0.09 | 0.31 | 2.03 | 1.76E-03 | 0.03 | 0.77 | 0.16 | 0.65 |
| 188 | PKHD1L1 | Fibrocystin-L | 1.83 | 0.01 | 0.07 | 1.61 | 0.05 | 0.23 | 1.13 | 0.47 | 0.80 |
| 189 | HMCN2 | Hemicentin-2 | 1.75 | 0.05 | 0.21 | 1.48 | 0.19 | 0.46 | 1.18 | 0.49 | 0.82 |
| 190 | IGDCC4 | Immunoglobulin superfamily DCC subclass member 4 | 1.61 | 0.13 | 0.37 | 2.30 | 1.81E-03 | 0.03 | 0.70 | 0.11 | 0.59 |
| 191 | GGH | Gamma-glutamyl hydrolase | 1.25 | 3.40E-03 | 0.06 | 1.09 | 0.38 | 0.62 | 1.15 | 0.03 | 0.38 |
| 192 | CRELD1 | Cysteine-rich with EGF-like domain protein 1 | 1.62 | 0.05 | 0.21 | 1.19 | 0.54 | 0.75 | 1.36 | 0.18 | 0.66 |
| 193 | FAM129B | Niban-like protein 1 | 2.38 | 0.04 | 0.19 | 2.72 | 0.01 | 0.10 | 0.88 | 0.66 | 0.88 |
| 194 | CHRDL1 | Chordin-like protein 1 | 1.39 | 0.05 | 0.21 | 1.31 | 0.13 | 0.40 | 1.07 | 0.64 | 0.87 |
| 195 | CDHR2 | Cadherin-related family member 2 | 1.71 | 0.01 | 0.10 | 1.39 | 0.16 | 0.43 | 1.23 | 0.24 | 0.70 |
| 196 | GLIPR2 | Golgi-associated plant pathogenesis-related protein 1 | 3.01 | 0.02 | 0.14 | 2.23 | 0.11 | 0.36 | 1.35 | 0.42 | 0.79 |
| 197 | PCDH18 | Protocadherin-18 | 1.77 | 0.01 | 0.07 | 1.58 | 0.05 | 0.23 | 1.12 | 0.49 | 0.82 |
| 198 | GP6 | Platelet glycoprotein VI | 3.58 | 0.02 | 0.15 | 3.18 | 0.05 | 0.23 | 1.13 | 0.77 | 0.92 |
| 199 | GKN1 | Gastrokine-1 | 2.71 | 6.16E-04 | 0.02 | 2.17 | 0.02 | 0.12 | 1.25 | 0.31 | 0.73 |
| 200 | CBLN4 | Cerebellin-4 | 4.98 | 0.01 | 0.10 | 3.09 | 0.08 | 0.31 | 1.61 | 0.32 | 0.73 |
| 201 | EHD3 | EH domain-containing protein 3 | 4.41 | 0.01 | 0.07 | 3.63 | 0.02 | 0.15 | 1.21 | 0.61 | 0.87 |
| 202 | ABRACL | Costars family protein ABRACL | 2.26 | 0.16 | 0.42 | 3.64 | 0.02 | 0.15 | 0.62 | 0.30 | 0.73 |
| 203 | CNTN3 | Contactin-3 | 3.76 | 0.01 | 0.07 | 3.53 | 0.01 | 0.08 | 1.07 | 0.84 | 0.94 |
| 204 | MRC2 | C-type mannose receptor 2 | 0.90 | 0.32 | 0.58 | 1.11 | 0.11 | 0.37 | 0.81 | 0.02 | 0.29 |
| 205 | CTSZ | Cathepsin Z | 1.43 | 4.16E-03 | 0.06 | 1.12 | 0.46 | 0.70 | 1.27 | 0.03 | 0.37 |
| 206 | CD300A | CMRF35-like molecule 8 | 1.56 | 0.20 | 0.46 | 0.63 | 0.32 | 0.57 | 2.48 | 0.02 | 0.32 |
| 207 | PCSK1N | ProSAAS | 3.66 | 0.04 | 0.21 | 3.81 | 0.04 | 0.23 | 0.96 | 0.94 | 0.98 |
| 208 | CD84 | SLAM family member 5 | 2.81 | 0.01 | 0.10 | 1.80 | 0.20 | 0.47 | 1.56 | 0.21 | 0.69 |
| 209 | GNPTG | N-acetylglucosamine-1-phosphotransferase subunit gamma | 1.19 | 0.03 | 0.17 | 1.15 | 0.09 | 0.32 | 1.03 | 0.57 | 0.85 |
| 210 | TRHDE | Thyrotropin-releasing hormone-degrading ectoenzyme | 1.85 | 0.02 | 0.14 | 1.27 | 0.44 | 0.68 | 1.46 | 0.12 | 0.62 |
| 211 | HPSE | Heparanase | 2.84 | 0.01 | 0.10 | 1.90 | 0.17 | 0.44 | 1.50 | 0.25 | 0.71 |
| 212 | TLN1 | Talin-1 | 0.99 | 0.92 | 0.97 | 1.39 | 1.17E-03 | 0.03 | 0.71 | 2.00E-03 | 0.14 |
| 213 | FN1 | Fibronectin | 1.00 | 0.78 | 0.90 | 0.96 | 4.16E-04 | 0.01 | 1.04 | 6.01E-04 | 0.12 |
| 214 | C4A | Complement C4-A | 1.58 | 0.29 | 0.56 | 0.37 | 0.10 | 0.34 | 4.24 | 0.01 | 0.28 |
| 215 | C1S | Complement C1s subcomponent (Fragment) | 1.09 | 0.77 | 0.89 | 0.52 | 0.07 | 0.29 | 2.10 | 0.04 | 0.38 |
| 216 | CP | Ceruloplasmin (Fragment) | 1.20 | 0.11 | 0.35 | 1.24 | 0.04 | 0.23 | 0.96 | 0.63 | 0.87 |
| 217 | PRG4 | Proteoglycan 4 (Fragment) | 1.06 | 0.05 | 0.21 | 1.02 | 0.62 | 0.80 | 1.04 | 0.19 | 0.66 |
| 218 | KRT2 | Keratin, type II cytoskeletal 2 epidermal | 2.45 | 0.01 | 0.07 | 1.82 | 0.09 | 0.31 | 1.34 | 0.23 | 0.70 |
| 219 | APOC1 | Apolipoprotein C-I | 1.00 | 0.91 | 0.96 | 0.99 | 0.06 | 0.28 | 1.02 | 0.06 | 0.51 |
| HC, healthy control group; T2DM, type 2 diabetes mellitus group; DKD, diabetic kidney disease group. | | | | | | | | | | | |

**Supplementary Table S4.** Comparative analysis of different model constructions.

| **Model** | **Proteomics** | | **Lipidomics** | | **Multi-omics** | |
| --- | --- | --- | --- | --- | --- | --- |
|  | Internal Accuracy | Prediction Accuracy | Internal Accuracy | Prediction Accuracy | Internal Accuracy | Prediction Accuracy |
| SVM | 94.29% | 80.95% | 59.04% | 61.90% | <50.0% | <50.0% |
| SVM-Selected | 82.86% | 61.90% | 59.04% | 59.04% | 93.33% | 80.95% |
| CNN | 100.00% | 85.71% | 93.33% | 71.43% | 100.00% | 71.43% |
| CNN-Selected | 85.71% | 71.43% | 100.00% | 85.71% | 100.00% | 90.48% |

SVM-Selected, the SVM model built based on the selected feature data; CNN-Selected, the CNN model built based on the selected feature data.

**Supplementary Table S5.** Four different proteins validated set by ELISA.

| **No.** | **Entry** | **Genes** | **Descriptions** | ***P* value** | | |
| --- | --- | --- | --- | --- | --- | --- |
|  |  |  |  | **HC/DM** | **HC/DKD** | **DM/DKD** |
| 1 | Q01518 | CAP1 | Adenylyl cyclase-associated protein 1 | - | ↓** | ↓* |
| 2 | P29350 | PTPN6 | Tyrosine-protein phosphatase non-receptor type 6 | ↓** | ↓** | - |
| 3 | Q99439 | CNN2 | Calponin-2 | - | ↓* |  |
| 4 | Q9Y490 | TLN1 | Talin-1 | - | ↓* | ↓** |

“↑”, up; “↓”, down. “-”, no statistical significance. (*: *p*＜0.05, **: *p*＜0.01). HC, healthy control group; DM, type 2 diabetes mellitus group; DKD, diabetic kidney disease group.

**Supplementary Table S6.** Eight different lipids of the validation set by lipidomics.

| **No.** | **Ret.T** | **m/z** | **Metabolites** | **Molecular Formula** | **Fold Change** | | |
| --- | --- | --- | --- | --- | --- | --- | --- |
|  |  |  |  |  | **T2DM/HC** | **DKD/HC** | **DKD/**  **T2DM** |
| 1 | 6.69 | 349.2345 | 17α,21-Dihydroxypregnenolone | C21H32O4 | - | [↓**](http://www.genome.jp/dbget-bin/www_bget?C13828) | ↓* |
| 2 | 6.78 | 368.2828 | Tetrahydrodeoxycorticosterone | C21H34O3 | ↑** | [↑**](http://www.genome.jp/dbget-bin/www_bget?C13828) |  |
| 3 | 7.80 | 546.3274 | LysoPC(P-18:0/0:0) | C26H54NO6P | - | - | ↓* |
| 4 | 10.80 | 465.3611 | Trihydroxycoprostanoic acid | C28H48O5 | **↑**** | **↑**** | ↑** |
| 5 | 12.15 | 726.5835 | GlcCer(d18:1/-18:1) | C42H79NO8 | ↑** | [↑**](http://www.genome.jp/dbget-bin/www_bget?C13828) | - |
| 6 | 14.19 | 555.5411 | Cer(d18:1/16:0) | C34H67NO3 | ↑** | [↑**](http://www.genome.jp/dbget-bin/www_bget?C13828) | ↑** |
| 7 | 14.17 | 603.4747 | FAHFA(18:1/13-O-18:0) | C36H68O4 | ↑* | - | - |
| 8 | 14.73 | 271.2228 | 3-Oxohexadecanoic acid | C_16_H_30_O_3_ | ↓* | - | - |

“↑”, up; “↓”, down. “-”, no statistical significance. (*: *p*＜0.05, **: *p*＜0.01). HC, healthy control group; T2DM, type 2 diabetes mellitus group; DKD, diabetic kidney disease group.
